# New insights into the heat responses of grape leaves via combined phosphoproteomic and acetylproteomic analyses

**DOI:** 10.1038/s41438-019-0183-x

**Published:** 2019-09-01

**Authors:** Guo-Tian Liu, Jian-Fu Jiang, Xin-Na Liu, Jin-Zhu Jiang, Lei Sun, Wei Duan, Rui-Min Li, Yi Wang, David Lecourieux, Chong-Huai Liu, Shao-Hua Li, Li-Jun Wang

**Affiliations:** 1 Beijing Key Laboratory of Grape Science and Enology and Key Laboratory of Plant Resources, Institute of Botany, the Chinese Academy of Sciences, Beijing, 100093 China; 2 Zhengzhou Fruit Research Institute, Chinese Academy of Agricultural Sciences, Zhengzhou, 450009 China; 3 College of Horticulture, Northwest A&F University, Yangling, 712100 China; 4 Universite´ de Bordeaux, ISVV, Ecophysiologie et Ge´nomique Fonctionnelle de la Vigne, UMR 1287, F-33140 Villenave d’Ornon, France; 5 INRA, ISVV, Ecophysiologie et Ge´nomique Fonctionnelle de la Vigne, UMR 1287, F-33140 Villenave d’Ornon, France; 6 University of Chinese Academy of Sciences, Beijing, 100093 China

**Keywords:** Abiotic, Protein sequencing

## Abstract

Heat stress is a serious and widespread threat to the quality and yield of many crop species, including grape (*Vitis vinifera* L.), which is cultivated worldwide. Here, we conducted phosphoproteomic and acetylproteomic analyses of leaves of grape plants cultivated under four distinct temperature regimes. The phosphorylation or acetylation of a total of 1011 phosphoproteins with 1828 phosphosites and 96 acetyl proteins with 148 acetyl sites changed when plants were grown at 35 °C, 40 °C, and 45 °C in comparison with the proteome profiles of plants grown at 25 °C. The greatest number of changes was observed at the relatively high temperatures. Functional classification and enrichment analysis indicated that phosphorylation, rather than acetylation, of serine/arginine-rich splicing factors was involved in the response to high temperatures. This finding is congruent with previous observations by which alternative splicing events occurred more frequently in grapevine under high temperature. Changes in acetylation patterns were more common than changes in phosphorylation patterns in photosynthesis-related proteins at high temperatures, while heat-shock proteins were associated more with modifications involving phosphorylation than with those involving acetylation. Nineteen proteins were identified with changes associated with both phosphorylation and acetylation, which is consistent with crosstalk between these posttranslational modification types.

## Introduction

Heat stress is a major factor in plant growth and productivity, and high temperature conditions are predicted to become more frequent worldwide^1^. Plants have evolved complex mechanisms to survive heat stress, including physiological, biochemical, cellular, and molecular processes^2,3^. Many studies have focused on better understanding the effects of heat stress on plants via transcriptomic and proteomic analyses^4–6^, and numerous genes and proteins associated with stress responses have been identified. In addition, a range of posttranslational modifications (PTMs) have been linked to plant stress responses^7–9^. Of these modification, perhaps the most studied is protein phosphorylation, which influences metabolism, transcription, translation, proteolysis, homeostasis, and signaling^10^, as exemplified by phosphoproteomic studies in plant species, such as *Arabidopsis thaliana*, maize (*Zea mays*), rice (*Oryza sativa*), soybean (*Glycine max*), and wheat (*Triticum aestivum*)^11–18^. Lys acetylation is another dynamic and reversible PTM that occurs on either the α-amino group at the N-terminus or the ε-amino group on the side chain of Lys residues^19^. Previous studies have indicated that protein acetylation regulates a wide range of cellular processes and phenomena, including transcription^20^, enzymatic activity^21^, protein interactions^22^, and protein stability^23^, as well as metabolic pathways^24^. Many Lys-acetylated proteins have been identified in both microbes and mammals, revealing roles in many cellular functions; however, the plant acetylproteome has been studied relatively little^25^.

Grape (*Vitis vinifera* L.) is an economically important crop species worldwide; however, its quality and yield are often constrained by heat stress^26,27^. Several studies have related changes in grape PTMs to stress responses. One example described a global comparative proteomic analysis of steady-state protein expression, as well as changes in phosphorylation and Lys acetylation of proteins from the mesocarp and exocarp of grape in response to infection by *Lobesia botrana*^7^. This analysis revealed 3059 proteins, 1135 phosphosites, and 138 Lys acetyl sites. In another study, hundreds of phosphoproteins were quantified in Cabernet Sauvignon grape leaves after abscisic acid (ABA) treatment^28^, and quantitative changes in the proteome and phosphoproteome of Barbera grapevine leaves after phytoplasma infection and recovery have been described. However, to our knowledge, the phosphoproteome and acetylproteome of grape plants subjected to abiotic stresses have not yet been described.

In a previous study, we analyzed transcriptomic and proteomic changes in leaves of grape plants grown under four different temperature regimes (25 °C, 35 °C, 40 °C, and 45 °C); uncovered cellular responses to sudden temperature changes; and characterized differentially expressed proteins, genes, and pathways^6^. Based on these results, in this study, we conducted phosphoproteomic and acetylproteomic analyses of the same material to reveal heat response PTMs in grape leaves.

## Results

### Protein phosphorylation and acetylation in grape leaves in response to high temperature

**Fig. 1:**
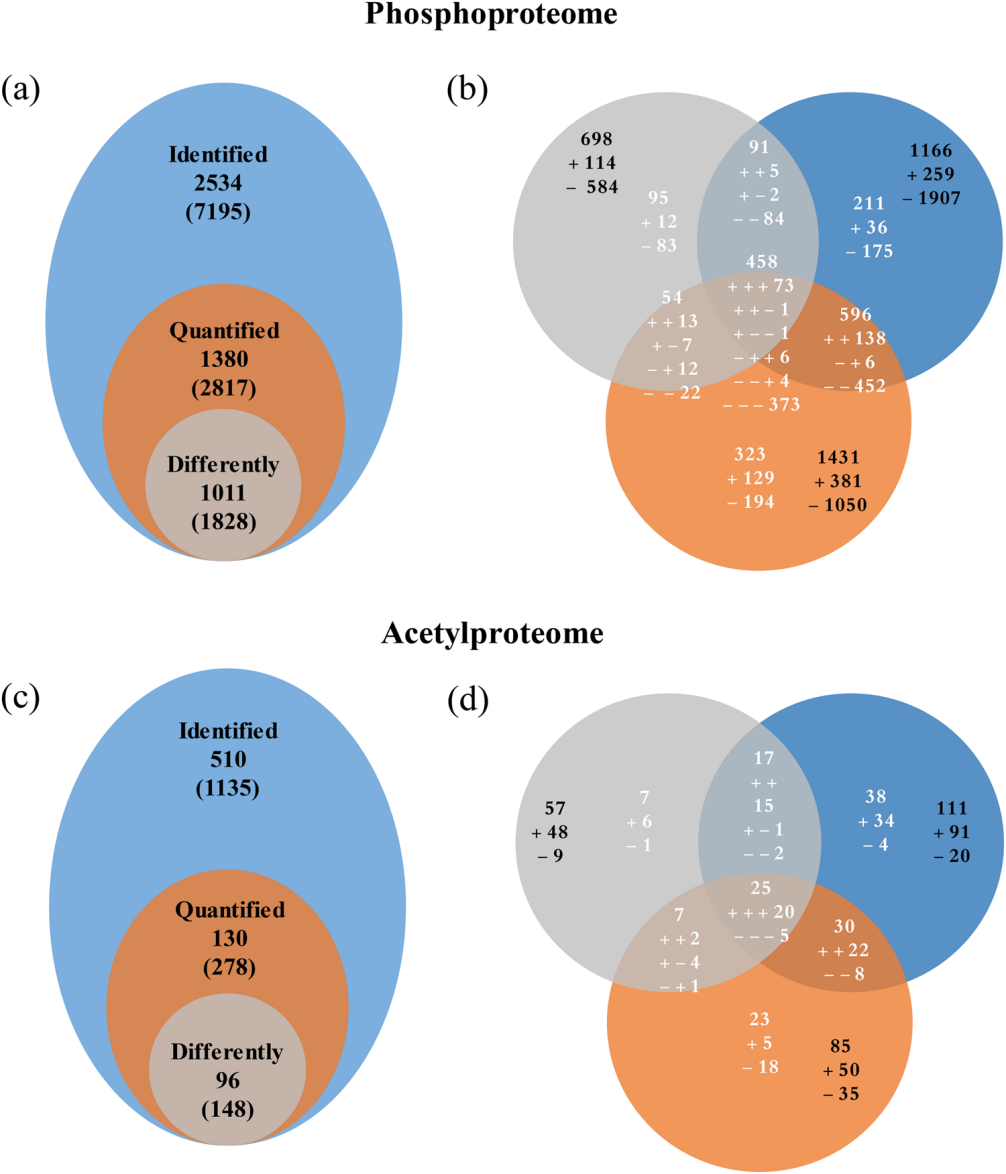
Overview of phosphoproteome and acetylproteome. The number of identified and quantified phosphoproteins and phosphosites (**a**) and acetyl proteins and acetyl sites (**c**) in grape leaves following exposure to temperatures of 35 °C, 40 °C, and 45 °C as well as differently changed protein phosphosites (**b**) and acetyl sites (**d**) at 35 °C, 40 °C, and 45 °C. The numbers outside of parentheses indicate phosphoproteins or acetyl proteins, and the numbers inside parenthesis indicate phosphosites or acetyl sites. The “ + ” and “−” indicate upregulated and downregulated sites, respectively, and the black numbers indicate the total number of upregulated or downregulated sites at 35 °C, 40 °C, and 45 °C

To gain insight into changes in protein phosphorylation and acetylation patterns under high temperature conditions, leaves from grape plants grown under four different temperature regimes (25 °C, 35 °C, 40 °C, and 45 °C) were collected, as we previously described^6^. The workflow is shown in Fig. S1. Peptides containing phosphorylation and Lys acetylation were quantified: a total of 2534 proteins with 7195 phosphosites were identified, and 1380 proteins with 2817 phosphosites were quantified. A total of 510 proteins with 1135 acetyl sites were ultimately identified, while 130 proteins with 278 acetyl sites were ultimately quantified (Fig. 1a, c).

**Fig. 2:**
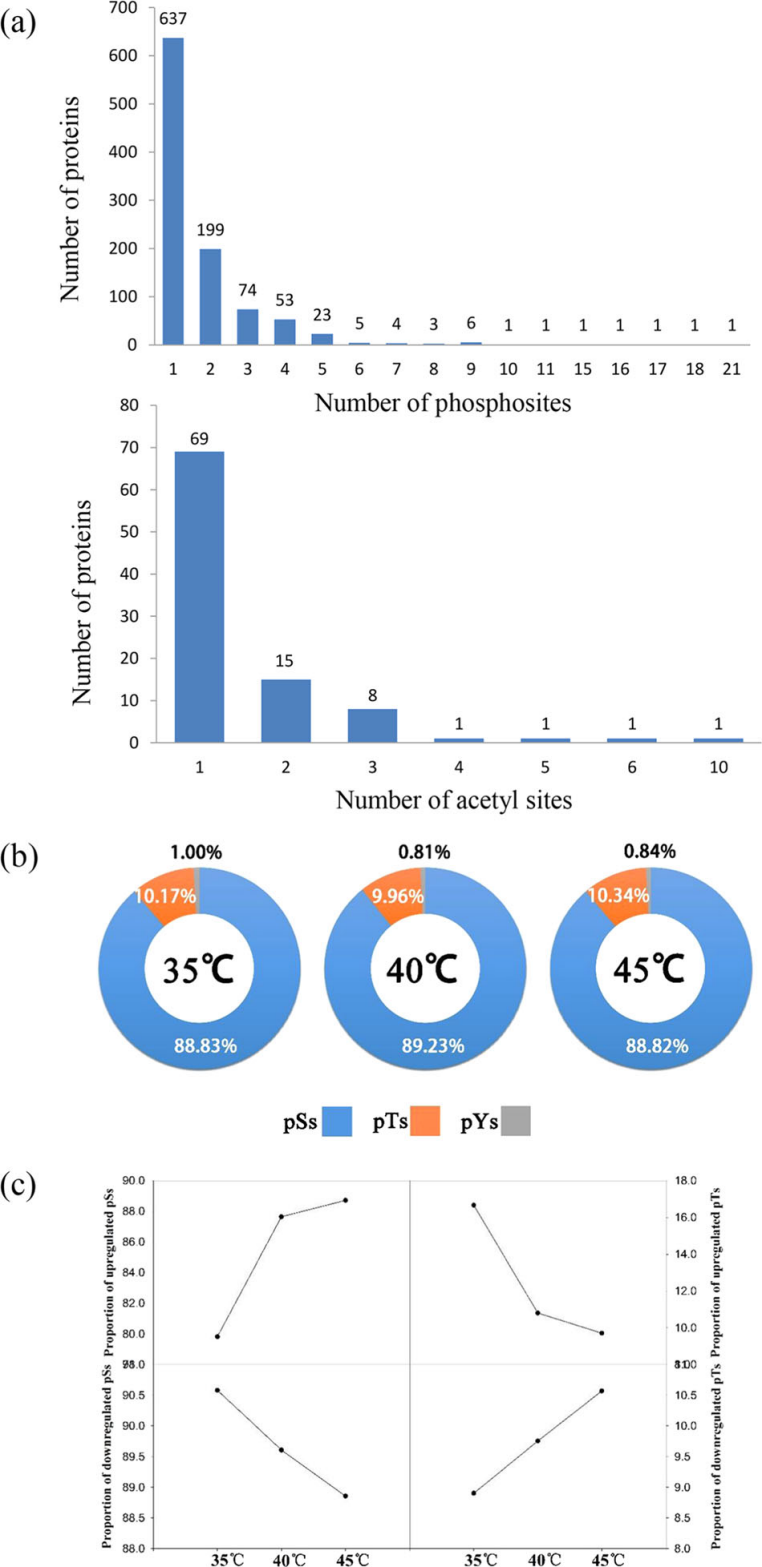
Summary of the phosphosites and acetyl sites in phosphoproteome and acetylproteome. The number of phosphoproteins according to the number of identified phosphosites and acetyl sites (**a**). Distribution of differently changed phosphosites at 35 °C, 40 °C, and 45 °C in grape leaves (**b**, **c**). pS phosphorylated serine, pT phosphorylated threonine, pY phosphorylated tyrosine

Differentially changed phosphosites (DCPSs) were then determined according to the following criteria: (1) phosphosites detected at all four temperatures; (2) the quantification ratio (35 °C/25 °C, 40 °C/25 °C or 45 °C/25 °C) > 1.3 or < 0.77; and (3) the phosphosite localization probability > 0.75. As a result, 1011 differentially changed proteins with 1828 DCPSs were identified (Fig. 1a). Among these DCPSs, 114, 259, and 381 phosphosites were found to be upregulated at 35 °C, 40 °C, and 45 °C, respectively, and 73 were coupregulated. There were five common upregulated phosphosites in the 35 °C and 40 °C samples, 13 in the 35 °C and 45 °C samples, and 138 in the 40 °C and 45 °C samples. At 35 °C, 40 °C, and 45 °C, 584, 1907, and 1050 phosphosites were downregulated, respectively, and 373 were codownregulated. In addition, there were 84 commonly downregulated phosphosites in the 35 °C and 40 °C samples, 22 in the 35 °C and 45 °C samples, and 452 in the 40 °C and 45 °C samples (Fig. 1b). We identified 637 proteins with 1 phosphosite, 199 proteins with 2 phosphosites, 74 proteins with 3 phosphosites, and 53 proteins with 4 phosphosites (Fig. 2a). The dominant phosphorylated amino acid was serine (~89%), followed by threonine (~10%). Only ~0.9% of the sites detected involved tyrosine (Fig. 2b). Moreover, we found that upregulated phosphosites involving serine (pSs) increased, while downregulated pSs decreased between the 35 °C and 45 °C samples; however, upregulated phosphosites involving threonine (pTs) decreased, and downregulated pTs increased between the 35 °C and 45 °C samples (Fig. 2c).

Using similar criteria to the above to identify differentially changed acetyl sites (DCASs), we identified a total of 96 proteins with 148 DCASs (Fig. 1c). In these DCASs, 48, 91, and 50 were upregulated at 35 °C, 40 °C, and 45 °C, respectively, compared with the control, while 20 were upregulated at all three temperatures. There were 15 commonly upregulated DCASs in the 35 °C and 40 °C samples, two in the 35 °C and 45 °C samples, and 22 in the 40 °C and 45 °C samples. In the 35 °C, 40 °C, and 45 °C treatments, 9, 20, and 35 DCASs were downregulated, respectively, and five were downregulated in all three samples. In addition, there were two downregulated DCASs in the 35 °C and 40 °C samples and eight downregulated DCASs in the 40 °C and 45 °C samples (Fig. 1d). Acetylation of these proteins was modified at Lys sites. There were 69 acetyl proteins with one acetyl site, 15 proteins with two acetyl sites, and 8 proteins with three acetyl sites (Fig. 2a).

### Sequence properties of phosphoproteins and acetyl proteins

**Fig. 3:**
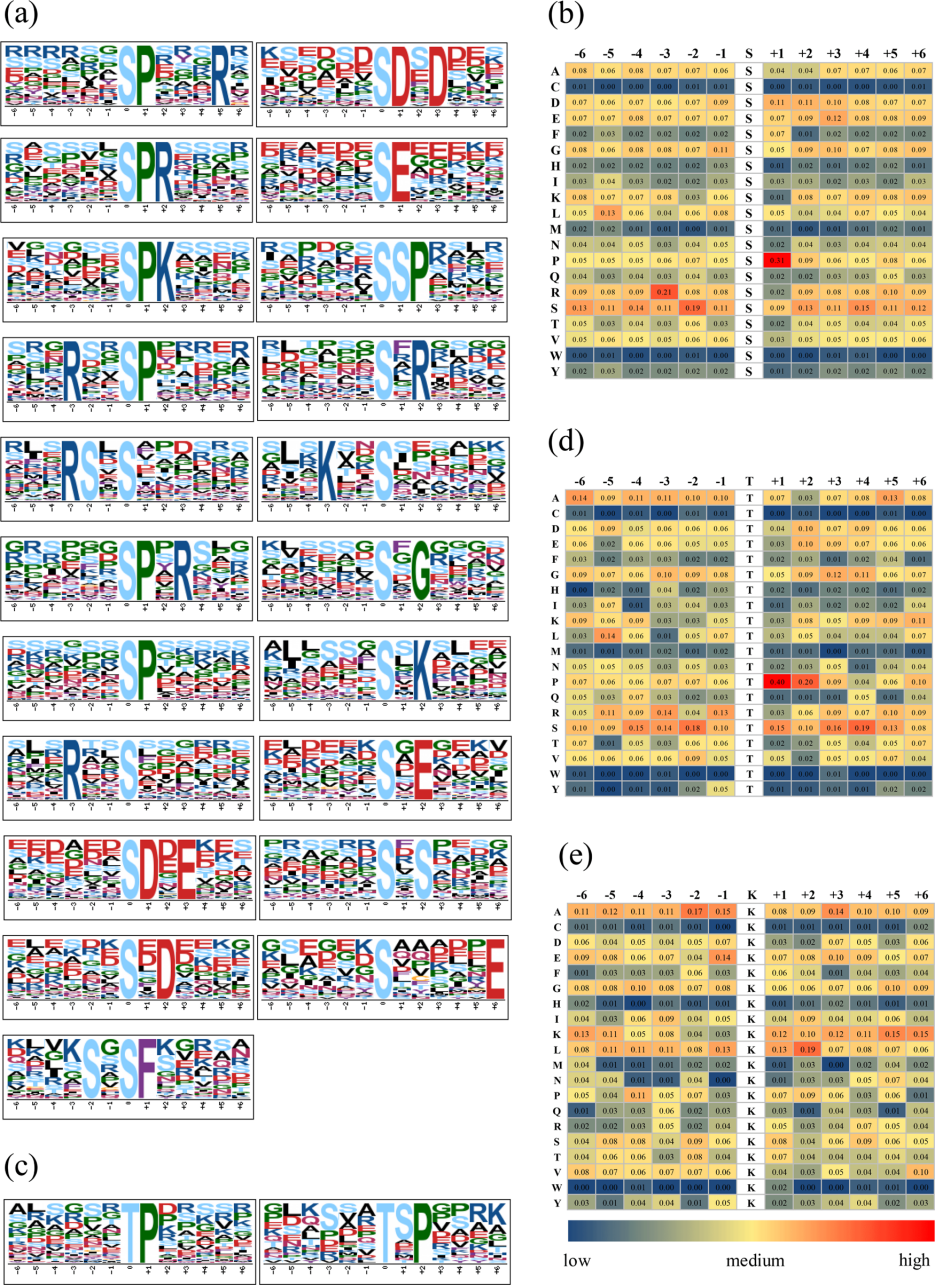
Motif-X analysis of the pSs, pTs and Kacs. (**a**, **c**): Graphical representation of the amino acid environment of the identified motifs. pS phosphorylated serine, pT phosphorylated threonine, Kac acetylated Lys. Upper scales of panels (**b**), (**d**) and (**e**): the position of the amino acids around the phosphosites (pSs, pTs) and acetyl sites (Kacs). Lateral scales of panels (**b**), (**d**) and (**e**): abbreviations of the twenty amino acids

To evaluate the sequence conservation around the identified phosphosites and acetyl sites, we used Motif-X software (http://motif-x.med.harvard.edu/) to compare the position-specific frequencies of the six flanking amino acid residues upstream and downstream from the sites. We identified 21 significantly enriched pS motifs from 1632 pSs and two pT motifs from 181 pTs (Fig. 3a, c). Due to limited pY (phosphorylated tyrosine) and Kac (acetylated Lys) sites, no motifs were identified for these positions. The frequency of different amino acids around the phosphosites was then determined, revealing the preferred residue for pSs to be P at + 1, R at -3, and S at -2. TP is known to be by far the most common pT motif^29^, and this was confirmed in our study (Fig. 3b, d, e). Moreover, we found that alanine (A), leucine (L), and glutamate (E) predominate at positions −2 to + 2 relative to Lys, which indicates that those amino acids may be conserved in grape protein acetylation sites.

### Functional classification and enrichment analysis of DCPSs and DCASs following exposure to high temperatures

**Fig. 4:**
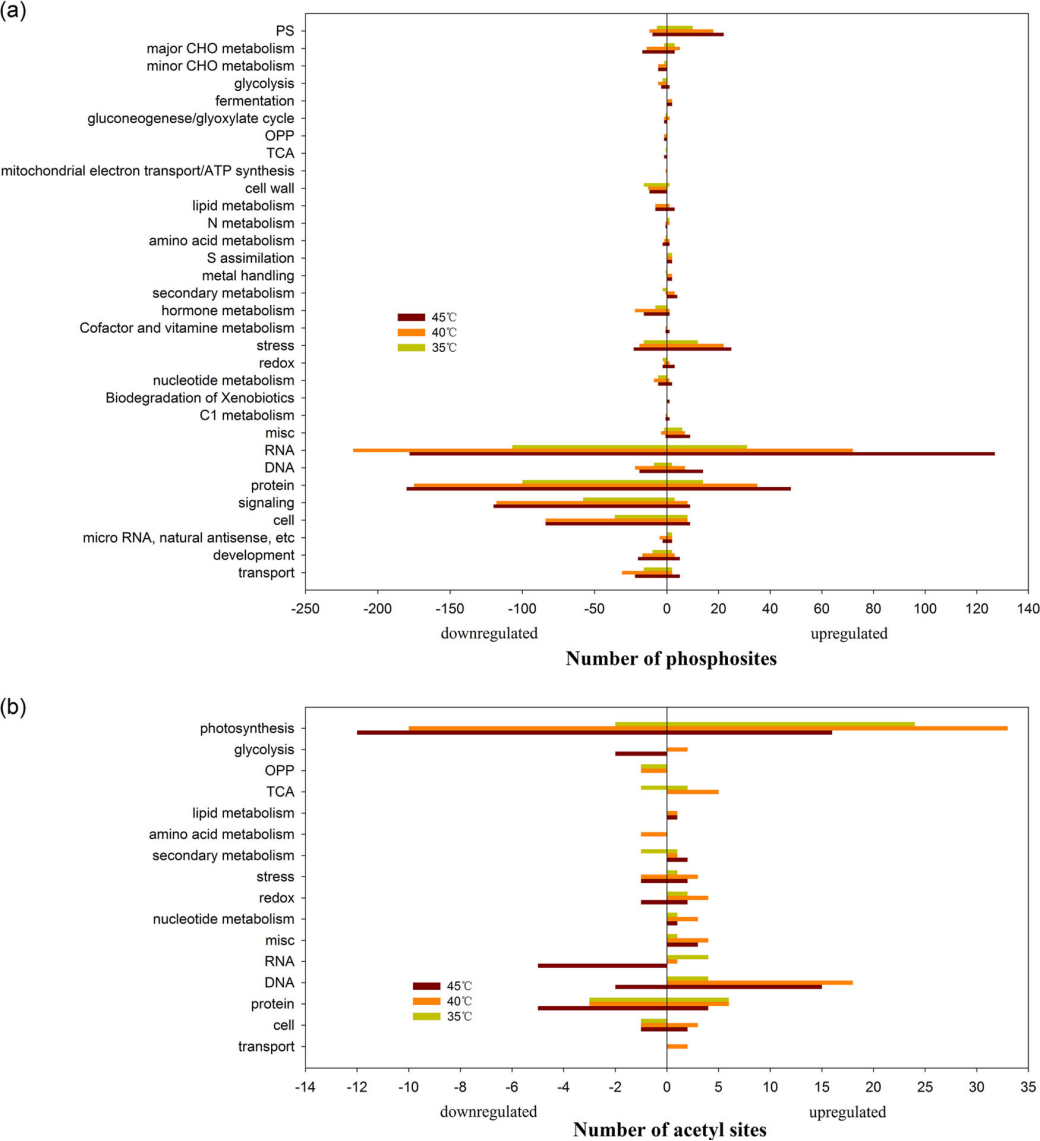
Functional categorization in phosphoproteome and acetylproteome. Main biological processes associated with proteins with differentially changed phosphosites (**a**) and acetyl sites (**b**) at 35 °C, 40 °C, and 45 °C in grape leaves. The *x*-axis indicates the number of upregulated (right) and downregulated (left) phosphosites

To functionally annotate the identified proteins, BLASTP (http://www.ncbi.nlm.nih.gov/BLAST/) was used to identify homologous proteins in TAIR database (http://www.arabidopsis.org/). Functional classification was then conducted using MapMan software (version 3.6.0RC1)^30^. The DCPSs were found to be mainly related to RNA regulation, protein synthesis, degradation, PTM, and signaling (Fig. 4a). Based on these analyses, we further analyzed proteins with up- or downregulated phosphosites in all 35 °C, 40 °C, and 45 °C samples. For 73 of these, 8 phosphosites were related to proteins involved in photosynthesis, including fructose-bisphosphate aldolase (FBA), ribulose bisphosphate carboxylase small chain 1a (RBCS1A), light-harvesting complex PSII subunit 6 (LHCB6), and photosystem II subunit R (PsbR). Twenty-three phosphosites were related to RNA, with 13 associated with RNA splicing factors. Eleven phosphosites were related to heat stress, such as heat-shock protein 23.6 (HSP23.6), HSP17.6, and CaM-binding protein 1 (Fig. S2). The 373 phosphosites that were downregulated in all three high-temperature samples were mainly associated with cells; PTM; synthesis and degradation of proteins; RNA transcriptional regulation; and signaling involving light, calcium and G-proteins (Fig. S3). For the 138 phosphosites unchanged at 35 °C but upregulated at 40 °C and 45 °C, seven were related to photosynthesis (including photorespiration), such as FBA, RuBisCO activase (RCA), peroxisomal (S)-2-hydroxy-acid oxidase (GLO1), and aminomethyltransferase (AMT). Forty-three phosphosites were associated with RNA processing, RNA regulation of transcription, and RNA binding, while 14 were SR-rich splicing factors, such as RS2Z33, RS41, and SCL30A. Ten phosphosites were related to heat stress, including HSP81.1, HSP81.2, HSP17.6, HSP23.6, HSP70.1, and CaM-binding protein 1 (Fig. S4). There were 129 phosphosites unchanged at 35 °C and 40 °C but upregulated at 45 °C, and these were mainly involved in RNA splicing and protein synthesis, degradation, and modification. There were only 13 phosphosites unchanged at 40 °C but upregulated at 35 °C and 45 °C, and they were mainly involved in RNA splicing (Fig. S5).

The DCASs were mainly related to photosynthesis, DNA synthesis, protein synthesis, degradation, and posttranslational modification (Fig. 4b). The 20 acetyl sites that were upregulated at all three relatively high temperatures were mainly associated with photosynthesis, such as PsbO2, RBCS1A, RBCL, phosphoglycerate kinase (PGK), and glyceraldehyde-3-phosphate dehydrogenase (GAPB) (Fig. S6). The five acetyl sites that were downregulated at all three relatively high temperatures were mainly associated with protein synthesis and degradation. Of the 22 acetylated proteins that were unchanged at 35 °C but upregulated at 40 °C and 45 °C, ten were related to DNA synthesis, and three were related to photosynthesis, including ribose 5-phosphate isomerase (Rib5P), RBCS1A, and PsbQ2 (Fig. S7). Five acetyl sites related to DNA synthesis, such as HTA11, were unchanged at 35 °C and 40 °C but upregulated at 45 °C, while 14 mainly related to photosynthesis, such as RCA, GAPB, glycolate oxidase, RBCS1A, and ribulose-phosphate 3-epimerase (RPE), were unchanged at 45 °C but upregulated at 35 °C and 40 °C (Fig. S8). The 33 acetyl sites upregulated only at 40 °C were mainly related to photosynthesis, respiration and DNA synthesis, such as triose phosphate isomerase (TIM), FBA, PGK, RCA, RBCS1A, PsaH2, PsbP1, PsbQ2, carbonate dehydratase, and HTB11 (Fig. S9).

### Integration of the phosphoproteomic and acetylproteomic data

Out of 1011 phosphoproteins and 96 acetyl proteins exhibiting changes in response to high temperatures, there were 19 with changes in both modifications (Table S1). Eight of these proteins were related to photosynthesis or photorespiration. LHCB6 phosphorylation (T180) and acetylation (K219 and K327) levels were upregulated at 35 °C, but showed no differential change or were downregulated at 40 °C and 45 °C. LHCB6 phosphorylation (T188) was upregulated at all three high temperatures. The acetylation (K175 and K177) of RBCL was upregulated at 35 °C, 40 °C, and 45 °C, while phosphorylation (Y190 and S228) was upregulated only at 45 °C. The acetylation levels (five sites: K183, K201, K227, K236, and K314) of RBCS1A were upregulated at 35 °C, 40 °C, and 45 °C; however, the phosphorylation levels of this protein (four sites: T151, Y153, S117, and S180) were upregulated only at 45 °C. RCA acetylation increased significantly in response to all three high temperatures. Histone H2A.6 (HTA1) and H2A.4 are associated with DNA synthesis and chromatin structure. The acetylation level of H2A.6 was upregulated at 35 °C, while the phosphorylation level was downregulated at all three temperatures. The acetylation level of H2A.4 was downregulated at 45 °C, while the phosphorylation level was upregulated at 35 °C and 45 °C (Table S1).

## Discussion

### Phosphorylation, rather than acetylation, of SR-rich splicing factors is involved in the upregulation of alternative splicing (AS) genes in response to high temperatures in grape leaves

**Fig. 5:**
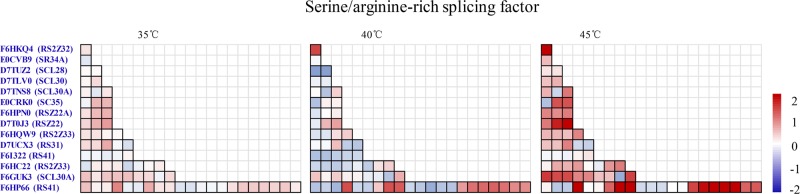
Phosphorylation level change of SR-rich splicing factors at 35 °C, 40 °C and 45 °C in grape leaves. Each block represents a phosphosite, and the different colors of each block represent up- or downregulation of the phosphorylation level

Alternative splicing involves the formation of two or more different transcripts from the same pre-mRNA molecule. The identity of the splice sites is largely determined by RNA-binding proteins, most notably SR proteins and heterogeneous nuclear ribonucleoproteins, which promote and inhibit binding of the spliceosome, respectively^31^. The precise formation of mRNA splice variants is determined by splicing factors that define the exon–intron boundaries between. SR proteins modulate alternative splicing via concentration-dependent and phosphorylation-dependent splice site selection, while the activity and localization of SR proteins are regulated by phosphorylation^32^. In plants, notable changes in alternative splicing are triggered by a wide variety of abiotic stresses^33–35^. A subset of human SR proteins comprise nucleocytoplasmic shuttling proteins with diverse roles in aspects of postsplicing, such as mRNA export, stability, and mRNA translation^36^. In our previous study, we observed a rapid change in pre-mRNA splicing in grape leaves at 35 °C, as 206 differential splicing events were detected, whereas 1075 and 748 were detected at 40 °C and 45 °C, respectively^6^. Moreover, we found that the abundance of SR45, SR30, and SR34 in grape leaves increased gradually as ambient temperatures increased^6^. These results are consistent with additional alternative splicing events that occur in response to high temperatures in grape leaves. We also found that the phosphorylation levels of 11 SR-rich splicing factors were mainly upregulated at 35 °C, 40 °C or 45 °C, which included RS2Z32, SR34A, SCL30, SCL30A, SC35, RSZ22A, RSZ22, RS31, and RS41. However, the downregulated phosphosites at each high temperature were associated only with SCL28. These results indicated that phosphorylation upregulation of SR-rich splicing factors is important for promoting alternative splicing during the response to high temperatures in grape leaves (Fig. 5; Supplementary Table S2). Some studies have also demonstrated that SCL30A, SR34, RS2Z33, and RS41 are involved in plant responses to salinity or high/low temperatures^37–39^. The increase in the phosphorylation level of these SR-rich splicing factors would strengthen their own activities and promote alternative splicing, which further explains why increased numbers of alternative splicing events occurred in grape leaves in response to high temperature. However, SR proteins did not appear to be acetylated during the grape leaf response to high temperature. Therefore, we conclude that phosphorylation, rather than acetylation, of SR-rich splicing factors is involved in the upregulation of alternative splicing during the grape leaf response to high temperatures.

### Acetylation modulates more photosynthesis proteins and is more sensitive to high temperatures than is phosphorylation during responses to high temperatures in grape leaves

**Fig. 6:**
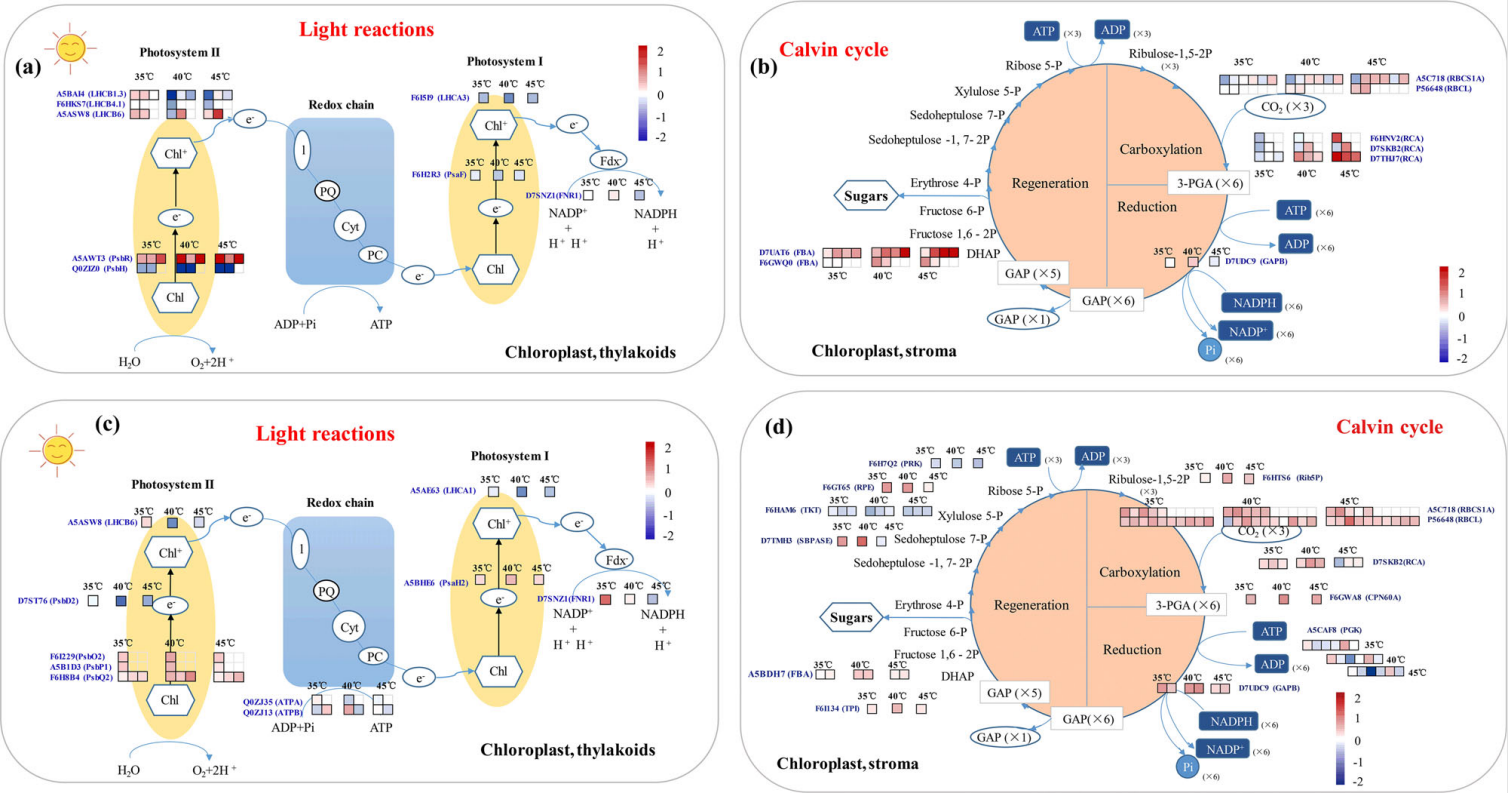
Schematic representation of phosphoprotein or acetylproteins involved in photosynthesis. Changes in phosphorylation levels (**a**) and acetylation levels (**c**) of proteins related to the light reactions of photosynthesis at 35 °C, 40 °C, and 45 °C in grape leaves. Changes in phosphorylation levels (**b**) and acetylation levels (**d**) of proteins related to the Calvin cycle of photosynthesis at 35 °C, 40 °C, and 45 °C

Photosynthesis involves highly heat-sensitive enzymes, and previous studies have shown that phosphorylation of photosynthesis proteins plays an important role in regulating photosynthesis^40,41^. The photosynthetic light reactions are catalyzed by thylakoid-embedded pigment–protein complexes (PSII, Cytb6f, and PSI), while the photooxidation of water during the light reactions of photosynthesis involves proteins of the oxygen-evolving complex (OEC), which are termed PsbO, PsbP, and PsbQ. In addition, PsbR has an auxiliary role in PsbP binding to PSII. In this study, we found that the phosphorylation levels of PsbR (at phosphosites Y52, S67, and Y77) at 35 °C, 40 °C, or 45 °C were upregulated (Fig. 6a; Table S3a). Furthermore, the acetylation levels of all oxygen-evolving complex components (PsbQ2, PsbP1, and PsbO2) were upregulated at 35 °C, 40 °C, or 45 °C, while their phosphorylation levels did not change (Fig. 6c). These results indicated that the oxygen-evolving complex may utilize acetylation and not phosphorylation to coordinate the response to high temperatures in grape leaves.

The photosystem II (PSII) core proteins D1 and D2, as well as the inner antenna protein CP43 and the minor PSII subunit PsbH, are phosphorylated, mostly by the kinase STN8, at Thr residues^42–44^. In this study, we found that the phosphorylation levels of PsbH (phosphosites T3 and T5) in grape leaves at 35 °C, 40 °C, or 45 °C were downregulated (Fig. 6a), and that the acetylation levels of PsbD2 at 35 °C were downregulated (Fig. 6c). The phosphorylation levels of PsaF and the acetylation levels of PsaH2 at the three high temperatures were upregulated (Fig. 6a, c). These results constitute the first report indicating that the acetylation of PSI proteins represents a response to high temperature in plants.

The light-harvesting proteins LHCB1, LHCB2, and LHCB4 are phosphorylated by the STN7 kinase^42,45^. In our study, we found that the phosphorylation levels of LHCB1 and LHCB6 were upregulated, but the phosphorylation levels of LHCB4 were downregulated in grape leaves in response to high temperatures (Fig. 6a). In addition to phosphorylation, LHC proteins undergo other PTMs such as Lys acetylation^46,47^. Acetylation can have a range of biochemical and biological effects, including the determination of enzyme activity, alteration of protein stability and modification of protein–protein interactions^48–50^. As an example, acetylation of LHCB1 and LHCB2 appears to influence LHC attachment to PSII complexes: the peripheral LHC antenna that is more loosely bound to PSII shows more extensive Lys acetylation than do PSII–LHCII supercomplexes^47^. In our study, the acetylation level of LHCB6 at high temperatures was downregulated or unchanged in grape leaves (Fig. 6c; Supplementary Table S3b). In addition, the phosphorylation level of LHCA3 and the acetylation level of the LHCA1 protein at high temperatures were downregulated in grape leaves (Fig. 6a, c; Supplementary Table S3a, b). These results indicated that the light-harvesting proteins of PSII and PSI responded to the phosphorylation or acetylation levels in the grape leaves in response to high temperatures.

RuBisCO is the first enzyme in carbon fixation, and it exists as a holocomplex of eight small subunits (RBCS) and eight large subunits (RBCL) that contain multiple phosphosites^51,52^ and are reversibly phosphorylated in many plant species^52,53^. RCA is an ancillary photosynthesis protein essential for RuBisCO activity. It has been reported that phosphorylation of RuBisCO results in a substantial increase in its activity, while desphosphorylation results in a major decrease in its activity^54^. However, the phosphorylation of RCA may result in decreased activity of RuBisCO^40,41^. In our study, the phosphorylation levels and phosphosites of RBCL and RBCS1A increased at high temperatures (Fig. 6b; Supplementary Table S3a), and the RCA showed the same trend as did RuBisCO. Based on these data, we concluded that the phosphorylation of RuBisCO may compensate for its decreased activity due to the phosphorylation of RCA. In addition, RuBisCO subunits undergo Lys acetylation in response to different light conditions^55^. The RuBisCO holocomplex has multiple Lys acetyl sites, which are located either in the RuBisCO catalytic center^46,56^, at the interface between the two RBCL subunits^46,57^, or at a location that is important in defining the RuBisCO tertiary structure^57^. Lys acetylation is thought to affect RuBisCO activity, as well as interactions between subunits and other molecules, and recent studies have shown that Lys acetylation suppresses RuBisCO activity^46,55^. In this study, almost all of the identified acetyl sites of RuBisCO (RBCL and RBCSA1) and RCA were upregulated at different high temperatures compared with the control (Fig. 6d; Supplementary Table S3b). Thus, acetylation and phosphorylation of RuBisCO and RCA may jointly help coordinate the light reactions and carbon assimilation in response to the carbon status of the cell under different high temperatures. Moreover, acetylation may have a larger effect on function rather than phosphorylation with respect to RuBisCO modification. In the green alga *Chlamydomonas reinhardtii*, RCA is phosphorylated at S53 by a thylakoid-localized kinase^58^. RCA is mainly localized in the stroma, but a small portion of the enzyme is associated with the thylakoid membrane^59^. It has been proposed that phosphorylation of RCA promotes its attachment to the membrane, thereby protecting Stt7 from proteolysis^58,60^. The relocation may also reduce the activity of RuBisCO under specific environmental conditions^58^. In Arabidopsis, RCA is phosphorylated at two sites, T78 and S172^41^, and in the dark, the proportion of phosphorylated T78 sites increases^61,62^. In this study, the phosphorylation levels of six sites of three RCA proteins (F6HNV2, D7SKB2, and D7THJ7) and the acetylation levels of three sites of one RCA protein (D7SKB2) were upregulated compared with the control in grape leaves under high temperature (Fig. 6b, d; Supplementary Table S3).

Other enzymes of the Calvin cycle have bene identified as being phosphorylated. Phosphoglycerate kinase (PGK) is known to be phosphorylated in Arabidopsis, rice, and maize;^53,61,63,64^ while the second two species have the same phosphosite, Arabidopsis PGK is phosphorylated in a domain near the N-terminus. GAPDH has several phosphorylation sites, but they differ considerably between different organisms; thus, it has been suggested that phosphorylation may not be a key factor in regulating GAPDH activity in chloroplasts^64^. It has been noted that PGK and GAPDH are also targets of Lys acetylation^46,65^, and the activities of both enzymes increase upon deacetylation. Thus, Calvin cycle enzymes are subject to complex regulation by PTMs, but the enzymes involved in such modifications have yet to be identified^64,66^. In our study, in addition to those of RuBisCO and RCA, the phosphorylation levels of FBA and GAPB increased at high temperatures (Fig. 6b; Supplementary Table S3a). Moreover, the acetylation levels of PGK, FBA, GAPB, RPE, and TIM increased under the heat treatments (Fig. 6d). However, the acetylation level of three sites of transketolase (TKT) decreased (Fig. 6d; Supplementary Table S3b). Therefore, both phosphorylation and acetylation are associated with the modulation of key enzymes of carbon assimilation in response to high temperatures (Fig. 6b, d). Moreover, it appears that the Calvin cycle may be affected more by acetylation than by phosphorylation. In addition, for all the investigated photosynthesis proteins in grape leaves, acetylation modification occurred largely at 35 °C, and phosphorylation modification was not prevalent (Fig. 6), which suggests that acetylation of grape leaf photosynthesis proteins is more influenced than phosphorylation is by high temperatures. We conclude that photosynthesis is fundamentally affected by high temperatures, and can be regulated by both phosphorylation and acetylation. Moreover, the acetylation of photosynthesis proteins was more sensitive to high temperatures than was phosphorylation.

### HSPs exhibit more extensive phosphorylation than acetylation in response to heat exposure in grape leaves

**Fig. 7:**
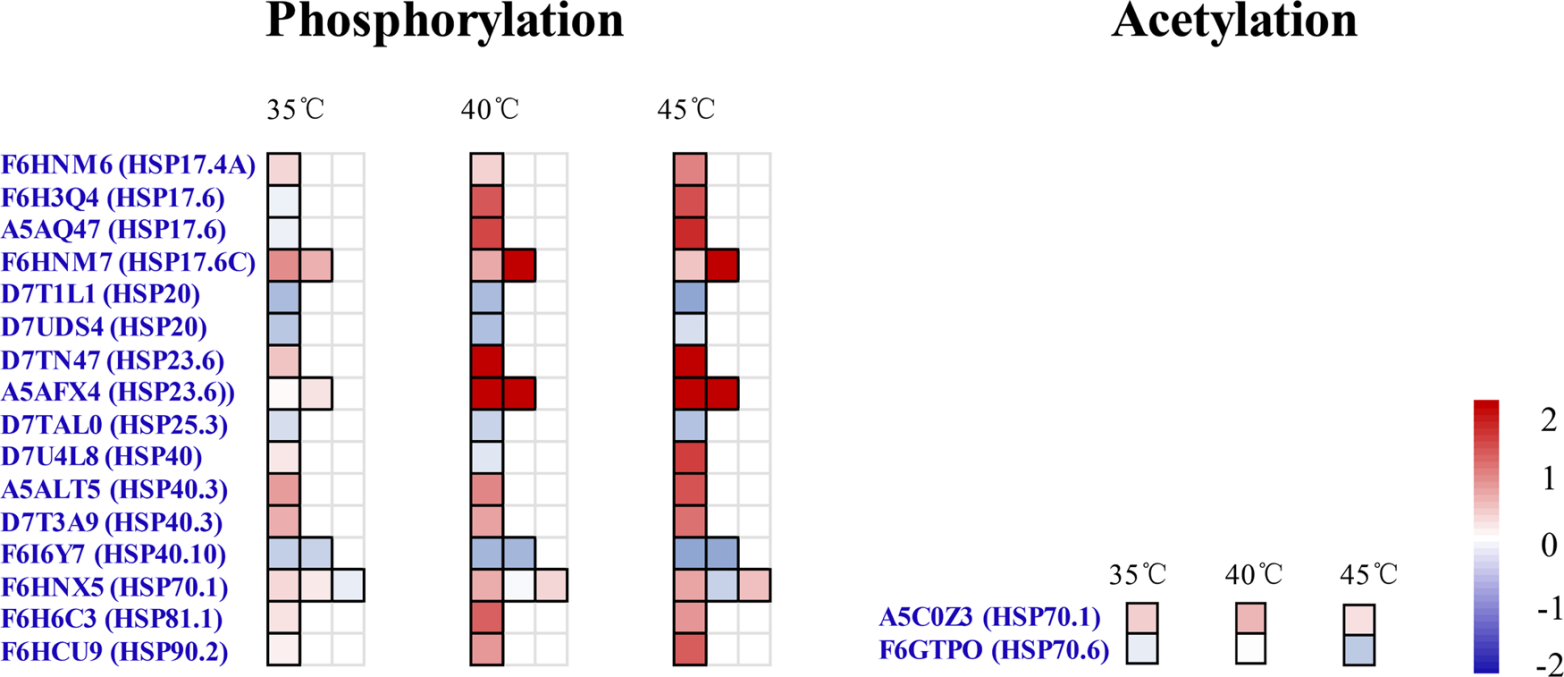
Phosphorylation and acetylation changes in heat shock protein (HSPs) at 35 °C, 40 °C, and 45 °C in grape leaves

Many studies have characterized large-scale responses to heat-shock responses in a variety of cells and organisms by the use of approaches, such as transcriptional profiling, differential displays, and proteomic analyses. HSPs act as molecular chaperones, reducing the aggregation and resolubilization of denatured proteins, promoting the folding of nascent polypeptides, and facilitating the refolding of denatured proteins^67–69^. In plants, HSPs can be classified into five groups based on molecular mass: small HSP (sHsp) proteins, chaperonins (GroEL and Hsp60), Hsp70 (DnaK) proteins, Hsp90 proteins, and members of the Hsp100 (Clp) family. Many studies have reported that high temperatures lead to increases in HSP levels in plants^70^. However, there are few studies on the acetylation and phosphorylation of HSPs in plants under heat or other stresses^71^. Characterized the phosphoproteome of different tissues of wheat cultivars that exhibit different degrees of drought tolerance following simulated drought and recovery. Notably, the phosphorylation levels of HSP60 and HSP90 were found to be upregulated in response to drought in the drought-tolerant cultivar. In another study^72^, reported the effects of a short-term and moderate increase in temperature on the wheat leaf and spikelet phosphoproteome, which involved a substantial increase in the abundance of S224 of HSP90 and S577 of HSP60-3A. The acetylation of HSP70 has been described as a regulatory mechanism that temporally balances protein refolding/degradation in response to stress^73,74^, while HSP20 phosphorylation in mammalian systems has been implicated in a variety of pathophysiological processes but most prominently in cardiovascular disease^75^. In this study, we found that the phosphorylation or acetylation levels of a number of HSPs were upregulated in grape leaves in response to heat treatment. Phosphorylation levels increased substantially at two sites (S10 and S154) of HSP17.6C (F6HNM7), one site (S12) at HSP23.6 (D7TN47), and two sites (S27 and S56) of HSP17.6 (A5AQ47) at three high temperatures (Fig. 7). The phosphorylation levels of other HSPs, including HSP17.4A, HSP17.6 (F6H3Q4), HSP17.6 (A5AQ47), putative HSP40, HSP40.3 (A5ALT5), HSP40.3 (D7T3A9), HSP81.1 (F6H6C3), and HSP81.2 (F6HCU9), were regulated at high temperatures. However, phosphorylation levels declined at one site of HSP20 (D7UDS4), one site of HSP20 (D7T1L1), and two sites of HSP40.10 (Fig. 7; Supplementary Table S4). The acetylation level increased at one site (K191) in HSP70.1 (A5C0Z3) and declined at another site (K613) in HSP70.6 (F6GTP0) (Fig. 7; Supplementary Table S4). In our previous study, 11 HSPs were coupregulated at both the transcriptional and translational levels in grape leaves upon exposure to the 40 °C and 45 °C treatments compared with the control, but not by low-temperature treatments; these proteins included HSP17.4, HSP17.6, HSP23.6, HSP40, HSP25.3, HSP70.1, and HSP81.1^6^. The results from this study indicate that these HSPs not only function in response to high temperatures by upregulating transcript and protein levels but also are involved in the heat tolerance of grape leaves via phosphorylation more so than acetylation. This study therefore underlines the importance of HSPs in grape thermotolerance.

### Crosstalk between protein phosphorylation and acetylation in response to high temperatures in grape leaves

Some proteins can undergo multiple modifications, and PTM crosstalk has recently been reported^76–80^. Xiong et al. found that an acetyl site (K324) on an enolase protein from rice was located next to a phosphosite (S325)^81^. In addition, the acetyl sites and phosphosites in two spliced isoforms of a gamma-interferon-inducible lysosomal thiol reductase precursor from rice were found to be in close proximity. Ahn et al. provided evidence for acetylation and phosphorylation crosstalk between two neighboring histone residues (S10 and K11) in vivo and in vitro in H2B (HIB4) in yeast^82^. Protein phosphorylation and acetylation have been associated with many cellular functions, as recently reported in *Mycoplasma pneumonia*^83^. In this study, 19 proteins exhibiting possible crosstalk between phosphorylation and acetylation were associated with the photosynthetic light reactions and the Calvin cycle, glycolysis, the TCA cycle, DNA and protein synthesis, cell division, and the cell cycle (Table S1). These results indicated that key proteins of some biological processes, especially photosynthesis, are regulated by concurrent phosphorylation and acetylation in grape leaves in response to high temperature.

Emerging evidence suggests that protein PTMs that are adjacent or in close proximity within a single protein often lead to regulation of protein function or are important in signaling^84^. Carlomagno et al. demonstrated that acetylation of Lys321 (contained within a KCGS motif) inhibited phosphorylation of Ser-324 in tau proteins^85^. Cook et al. also noted a similar competitive relationship between phosphorylation and acetylation of KIGS motifs^86^. Previous studies have shown that mutagenesis of the S113 and K230 sites of isocitrate dehydrogenase (Idh) results in reduced enzyme activity and substrate affinity, underlining the significance of both phosphorylation and acetylation in regulating Idh in *E. coli*^87,88^. Direct evidence of crosstalk between acetylation and phosphorylation was recently demonstrated in *M. pneumonia*^83^; however, there are no reports of phosphorylation and acetylation of the same protein in plants under stress. In this study, acetylation levels in a number of sites of RBCL increased in the grape leaves in response to 35 °C, 40 °C, and 45 °C, while the phosphorylation levels of a few sites increased only at 45 °C (Table S1). Acetylation levels at five sites and phosphorylation levels at two sites of RBCS1A increased in response to heat treatment (Table S1). These results indicated that RBCL may undergo different PTMs than does RBCS1A under high temperature in grape leaves and that their coordination modulates RuBisCO activity. Recent studies have indicated negative regulation of RuBisCO activity by Lys acetylation^46,55^, and we previously reported that RuBisCO activity significantly decreased in grape leaves under 43 °C high temperature^24^. In this study, RCA appeared to exhibit more acetylation than phosphorylation (Table S1). Other studies have indicated that the activity of RCA decreases in many plant species under high temperature^89^. Therefore, upregulation of acetylation levels may be a factor in the decrease in RuBisCO and RCA activities in the grape leaf response to high temperatures. In addition, at different high temperatures, the phosphorylation and acetylation levels of proteins differed. For example, the LHCB6 phosphorylation (T180) and acetylation (K219 and K327) levels were upregulated at 35 °C, but did not change or were downregulated at 40 °C and 45 °C. Grape protein modification by acetylation appears to be as common as phosphorylation, and acetylation may provide a balance to phosphorylation in the regulation of protein activity. Congruent with previous studies in humans and microbes^83,87,88^, an upregulated acetyl site (K183) and an upregulated phosphosite (S180) are near one another in RBCS1A, and an upregulated acetyl site (K151) and a downregulated phosphosite (S154) are very close in a SAP domain-containing protein (E0CRG0). The regulatory mechanism of protein activity under high temperature is clearly very complex.

In summary, phosphoproteomic and acetylproteomic analyses were conducted on leaves of grape plants subjected to four different temperature regimes (25 °C, 35 °C, 40 °C, and 45 °C). The results revealed significant changes in phosphosites and acetyl sites, mainly following exposure to 40 °C and 45 °C. Phosphorylation, rather than acetylation, of SR-rich splicing factors was involved in the increase in AS events. Moreover, compared with phosphorylation modification, acetylation modification modulated more photosynthesis-related proteins and was more sensitive to high temperatures. Conversely, we conclude that modifications of HSPs during heat tolerance responses in grape leaves involve phosphorylation more than acetylation. We identified 19 proteins with significantly changed phosphorylation and acetylation levels, which is indicative of crosstalk between these PTMs. Acetylation may balance phosphorylation with regard to protein activity. Additional studies are needed to determine how key phosphorylation and acetylation proteins influence grape heat tolerance.

## Materials and methods

### Plant materials and heat treatments

The materials and treatments were identical to those of our previous study^6^. One-year-old “Jingxiangyu” grapevine (*Vitis vinifera* L.) cuttings were planted in pots and then grown in a greenhouse under 70–80% relative humidity and at 18 °C to 25 °C. When the sixth leaf (from the base to the apex) of each grapevine became mature, all the grapevines were divided into four groups and acclimated for 2 days in a controlled-environment chamber (70% average relative humidity, 25 °C/18 °C [12-h/12-h] day/night cycle). On day 3, the grapevines were subjected to the following treatments: (1) the plants of the control group were maintained at the optimal day/night temperature (25 °C/18 °C) in the abovementioned growth chamber; (2) the plants of the treatment groups were exposed to 35 °C, 40 °C, or 45 °C from 11:30 a.m. to 1:30 p.m. (the conditions were the same as those of the control, except for the temperature). The fourth to sixth leaves (from the base to the apex) of each plant were detached at 1:30 p.m. (the end of the heat stress treatment). Each biological replicate included three plants, and two replicates were used for the three treatments and for the control. The detached leaves were frozen in liquid nitrogen immediately and then stored at −80 °C for further analysis.

### Protein extraction

Proteins were extracted using the cold-acetone method and digested as described previously^90^, and tryptic peptides were incubated with a 4-plex iTRAQ labeling kit (114 for 25 °C, 115 for 35 °C, 116 for 40 °C, 117 for 45 °C)^90^.

### High-pH fractionation and the enrichment of phosphopeptides

High-pH reversed-phase high-pressure liquid chromatography (HPLC) was used for peptide fractionation on a Gilson 300 series system. A total of 4 mg of each desalted iTRAQ-labeled sample corresponding to each of the four temperature treatments was solubilized individually in 200 µL of 0.02% NH_4_OH (pH 10) and injected onto an XBridge column (Waters, C18 3.5 µm 2.1 × 150 mm) using a linear gradient of buffer B from 2-45% for 45 min (buffer A: 0.02% NH_4_OH, pH 10; buffer B: 90% acetonitrile, 0.02% NH_4_OH, pH 10). The fractions were collected for 1 min, after which 5% of each fraction was dried under vacuum and preserved at −80 °C for total proteome analysis after desalting with a StageTip + C18^91^. The remaining 95% of each fraction was combined, dried under vacuum and preserved at −80 °C for phosphoproteome analysis.

The metal affinity chromatography (IMAC) enrichment of phosphopeptides was adapted from the methods of Mertins et al.^91^. Ion-chelated IMAC beads were prepared from Ni-NTA Superflow agarose beads (Qiagen, MA). Nickel ions were removed with 50 mM EDTA, and the iron was chelated by passing the beads through an aqueous solution of 200 mM FeCl_3_, followed by three water washes and one wash with binding buffer (40% acetonitrile, 1% formic acid). iTRAQ-labeled reversed-phase (RP) fractions were solubilized in binding buffer and incubated with IMAC beads for 1 h. After three washes with binding buffer, the phosphopeptides were eluted with a 2x bead volume of 500 mM potassium hydrogen phosphate (pH 7.0), and the eluate was neutralized with 10% formic acid. The enriched phosphopeptides were then desalted using an Empore 3 M C18 (2215) StageTip prior to nanoLC-MS/MS analysis^92^.

### Enrichment of acetylpeptides

A mixture of iTRAQ-labeled samples corresponding to the four temperature treatments was desalted using a Sep-Pak tC18 cartridge (Varian, CA). Acetylpeptides were enriched using a PTMScan Acetyl-Lys Motif (Ac-K) antibody (#13416, Cell Signaling Technology, MA) according to the manufacturer’s instructions. The eluted peptides were desalted with an Empore 3 M C18 (2215) StageTip, dried and solubilized in 0.1% TFA/5% acetonitrile prior to nanoLC-MS/MS.

### Mass spectrometry identification

The enriched phosphopeptides or acetylated peptides were analyzed by nanoLC-MS/MS with an RSLC system connected to a Q Exactive mass spectrometer (ThermoFisher, San Jose, CA, USA). The samples were loaded onto a self-packed 100 µm × 2 cm trap packed with Magic C18AQ, 5 µm 200 A (Michrom Bioresources, Inc., Auburn, CA, USA) and washed with buffer A (0.2% formic acid) for 5 min at a flow rate of 10 µL min^-1^. The trap was switched in line with a custom-made analytical column (Magic C18AQ, 3 µm 200 A, 75 µm × 50 cm), and the peptides were fractionated at 300 nL min^-1^ with a multistep gradient (proteome: 4–15% buffer B (0.16% formic acid 80% acetonitrile) for 30 min, 15–25% B for 40 min, and 25–50% B for 45 min; acetylome: 4–25% buffer B for 35 min followed by 25–50% B for 65 min). Mass spectrometry data were acquired using Xcalibur (ThermoFisher, San Jose, CA, USA) in data-dependent acquisition mode with a cyclic series of a full scan acquired at a resolution of 120,000 followed by MS/MS scans (30% collision energy in the HCD cell) at a resolution of 30,000 of the 20 most intense ions and with a dynamic exclusion duration of 30 s.

### Phosphoproteomic and acetylproteomic data analyses

All the data were analyzed with MaxQuant proteomics data analysis workflow (version 1.6.0.1.) using the Andromeda search engine^93,94^. The type of LC-MS run was set to “Reporter ion MS2” with “iTRAQ4plex” as isobaric labels, and the reporter ion mass tolerance value was defined as 0.003 Da. The false discovery rate was set to 1% for protein, peptide spectrum match, and site decoy fraction levels. Peptides were required to have a minimum length of seven amino acids and a maximum mass of 4600 Da. MaxQuant was used to score fragmentation scans for identification based on a search with an allowed mass deviation of the precursor ion of up to 4.5 ppm. The allowed fragment mass deviation was 20 ppm. MS2 spectra were searched against the UniProt *Vitis vinifera* cultivar Pinot Noir/PN40024 database (downloaded on 01/16/2018) (https://www.uniprot.org/), which contained 29,907 entries combined with 262 common contaminants. Enzyme specificity was set as trypsin/P, which allowed three missed cleavages for phosphorylation data and five missed cleavages for acetylation data. Carbamidomethylation of cysteine was set as a fixed modification, and N-terminal protein acetylation and oxidation at methionine were set as variable modifications. The phospho (STY) and acetyl (K) parameters were set as variable modifications for the phosphorylation- and acetylation-enriched samples, respectively. The data are available via ProteomeXchange with identifier PXD014090.

The summed reporter ion intensity of each channel before enrichment was used as the normalization factor for phosphoproteomic or acetylproteomic quantitation. After filtering potential contaminants, a reverse localization score ≥0.75 for the phosphoproteome and acetylproteome was included in the data.

### Bioinformatic analysis

The differentially expressed proteins were functionally categorized based on MapMan ontology^30^. MapMan ontology analysis was carried out based on the BLASTP results using the *A. thaliana* predicted proteome as a reference. Functional categories were manually verified by literature searches, and proteins that were not associated with any biological process category were assigned to MapMan (Bin35). Motif-X was used for motif analysis, and the setting parameters for searching motifs included 20 occurrences and a significance of 0.000001. Functional interaction network analysis was performed using STRING software^95^, and the results were visualized by Cytoscape^96^.

## Supplementary information

Supplementary Figures and Tables
