# Supplementary material for: New insights into the heat responses of grape leaves via combined phosphoproteomic and acetylproteomic analyses
Source: Hortic Res. 2019 Sep 1;6:100. doi: 10.1038/s41438-019-0183-x (PMC6804945; doi:10.1038/s41438-019-0183-x)
Supplement: Supplementary file 1 — Supplementary Figures and Tables [file 41438_2019_183_MOESM1_ESM.docx]

**Supplementary Figures and Tables**

**
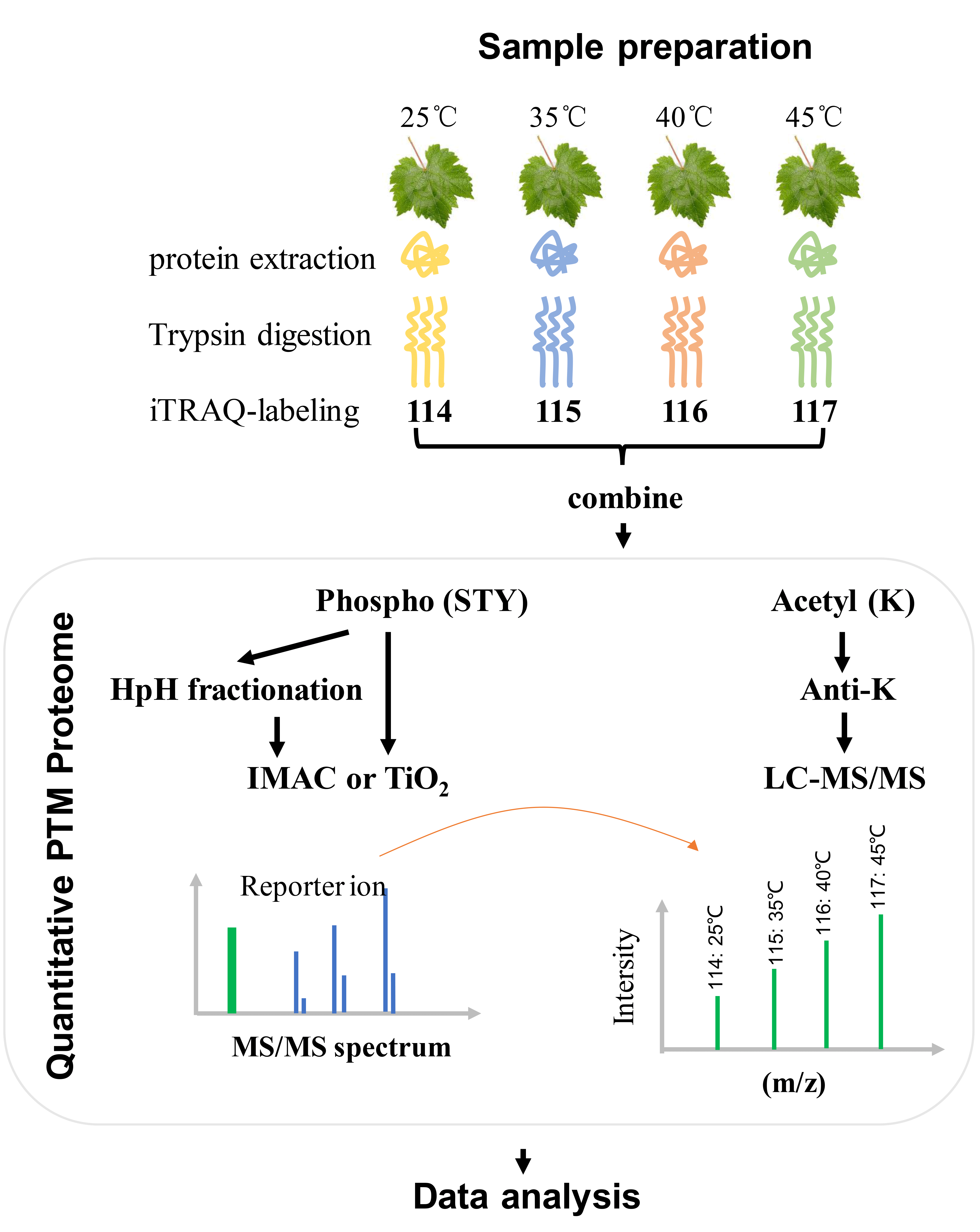
**

**Figure S1 Flow chart of phosphoproteome and acetylproteome analysis for grape leaves under different temperatures (25^o^C, 35^o^C, 40^o^C and 45^o^C)**

**
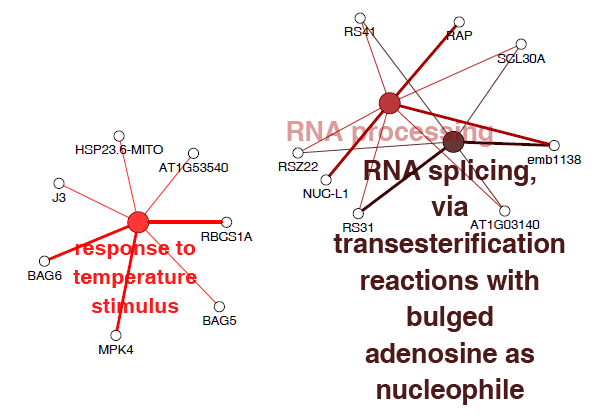
**

**Figure S2 The biology processes of proteins with co-upregulated phosophosites under 35^o^C, 40^o^C and 45^o^C compared to 25^o^C in grape leaves**

**
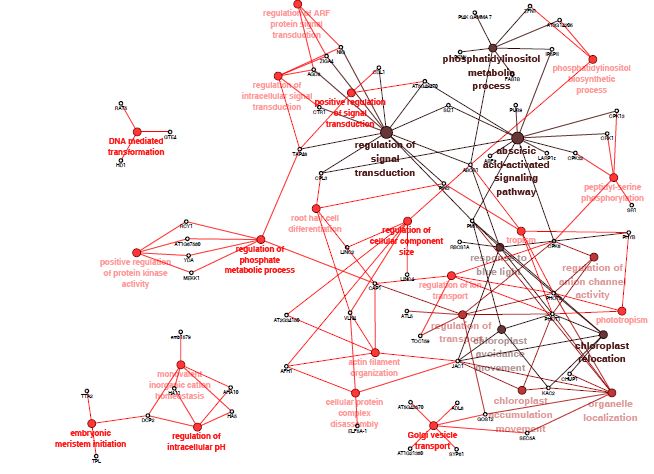
**

**Figure S3 The biology processes of proteins with phosophosites unchanged at 35^o^C but upregulated at 40^o^C and 45^o^C compared to 25^o^C in grape leaves.
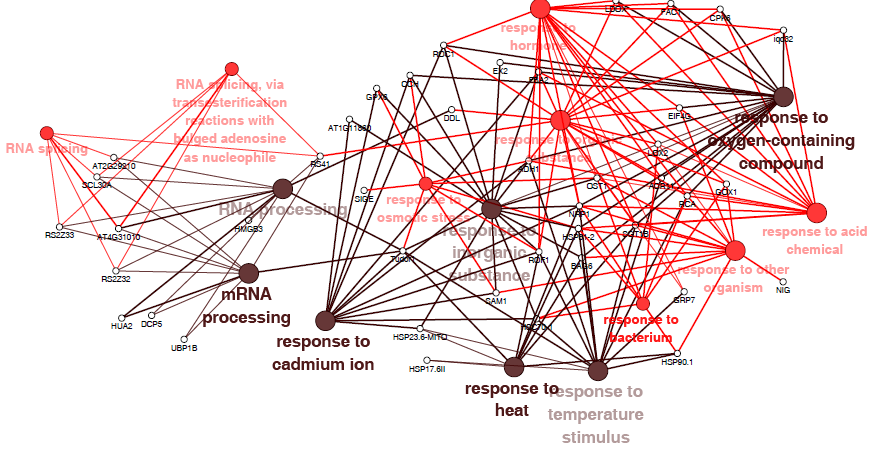
**

**Figure S4 The biological processes of proteins with phosphosites unchanged at 35^o^C but up-regulated at 40^o^C and 45^o^C in grape leaves**

**
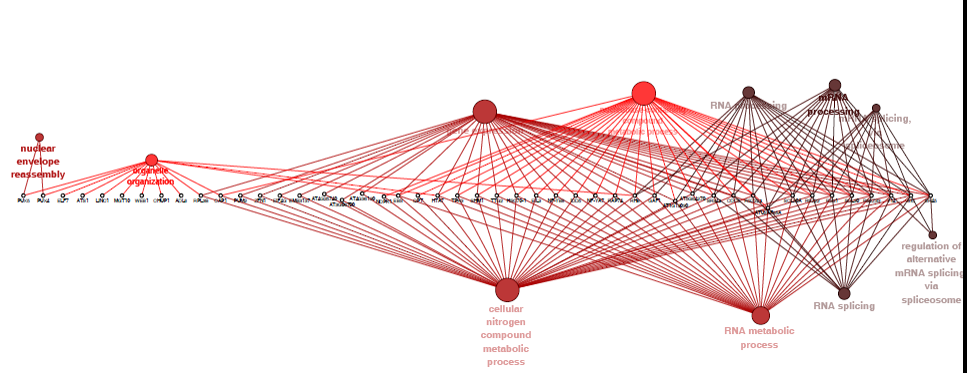
**

**Figure S5 The biology processes of proteins with phosophosites unchanged at 35^o^C and 40^o^C but upregulated 45^o^C compared to 25^o^C in grape leaves.**

**
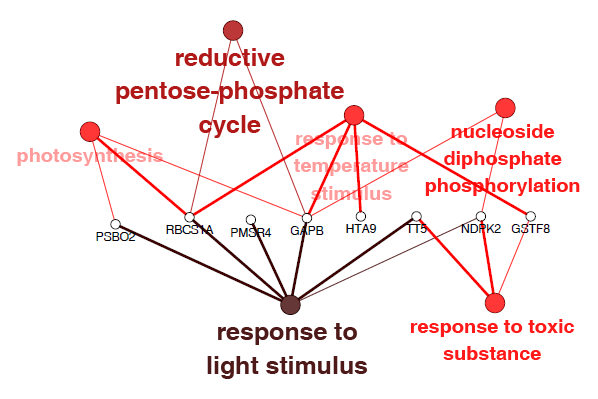
**

**Figure S6 The biology processes of proteins with co-upregulated acetylsites under 35^o^C, 40^o^C and 45^o^C compared to 25^o^C in grape leaves.**

**
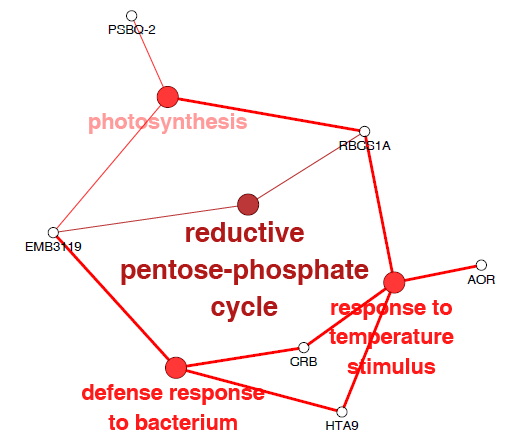
**

**Figure S7 The biology processes of proteins with acetylsites unchanged at 35^o^C but upregulated at 40^o^C and 45^o^C compared to 25^o^C in grape leaves.**

**
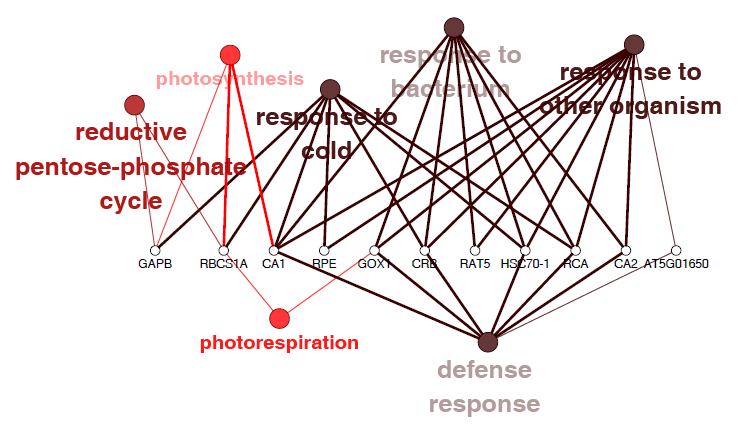
**

**Figure S8 The biology processes of proteins with acetylsites upregulatedat 35^o^C and 40^o^C but unchanged 45^o^C compared to 25^o^C in grape leaves.**  **
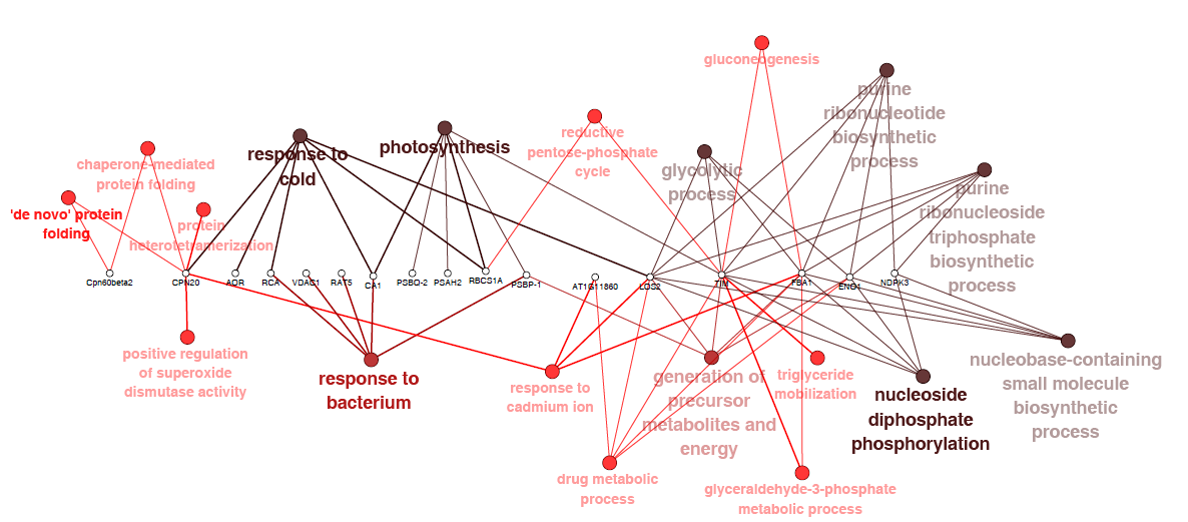
**

**Figure S9 The biology processes of proteins with acetylsites upregulatedat 35^o^C and 45^o^C but unchanged 40^o^C compared to 25^o^C in grape leaves.**

**Table S1 Phosphorylation and acetylation level changes in proteins identified in both phosphoproteome and acetylproteome under 35^o^C, 40^o^C and 45^o^C in grape leaves**

|  | **Uniprot ID** | **Protein name** | **Phosphosite /Acetylsites** | **35℃** | **40℃** | **45℃** |
| --- | --- | --- | --- | --- | --- | --- |
| **phosphorylation** | A5ASW8 | LHCB6 | T180 | 1.43 | 0.63 | 1.21 |
|  |  |  | T188 | 1.38 | 1.93 | 2.92 |
|  | D7SNZ1 | FNR1 | T169 | 0.99 | 1.12 | 0.67 |
|  | D7TKA1 | GLO1 | T161 | 1.19 | 2.09 | 1.97 |
|  |  |  | S215 | 1.22 | 1.42 | 1.77 |
|  | F6H7I9 | AMT | S339 | 1.20 | 1.41 | 1.30 |
|  | P56648 | RBCL | Y190 | 0.97 | 1.10 | 1.38 |
|  |  |  | S228 | 0.94 | 1.16 | 1.56 |
|  | A5C718 | RBCS1A | T36 | 0.55 | 0.56 | 0.58 |
|  |  |  | S117 | 0.90 | 0.88 | 1.54 |
|  |  |  | T151 | 1.06 | 1.26 | 1.49 |
|  |  |  | Y153 | 1.14 | 1.21 | 1.41 |
|  |  |  | Y177 | 0.83 | 0.75 | 0.85 |
|  |  |  | S180 | 1.31 | 1.32 | 1.48 |
|  | D7UDC9 | GAPB | T298 | 1.05 | 1.36 | 0.85 |
|  | D7SKB2 | RCA | S53 | 0.65 | 0.81 | 0.73 |
|  |  |  | S79 | 0.98 | 1.44 | 1.90 |
|  | F6GSG7 | GAPC2 | S205 | 0.60 | 0.61 | 0.96 |
|  | D7TWP2 | BCA1 | S106 | 0.96 | 0.88 | 0.74 |
|  | F6HHP1 | ADA2a | S200 | 0.83 | 0.75 | 0.72 |
|  |  |  | S358 | 0.95 | 0.88 | 0.76 |
|  |  |  | S460 | 1.02 | 0.68 | 0.69 |
|  | E0CRG0 | SAP domain- containing protein | S154 | 0.74 | 0.46 | 0.43 |
|  |  |  | S162 | 0.97 | 0.83 | 0.71 |
|  |  |  | S417 | 0.95 | 0.82 | 0.60 |
|  |  |  | T532 | 0.74 | 0.46 | 0.50 |
|  |  |  | T536 | 0.67 | 0.35 | 0.41 |
|  |  |  | S555 | 0.72 | 0.62 | 0.73 |
|  |  |  | S558 | 1.09 | 1.39 | 1.24 |
|  | A5BE97 | Probable  histone H2A.4 | S138 | 1.36 | 1.14 | 1.91 |
|  | E0CT43 | HTA1 | S129 | 0.61 | 0.37 | 0.59 |
|  | E0CRN7 | RPS6B | S229 | 0.43 | 0.16 | 0.22 |
|  |  |  | S231 | 0.39 | 0.13 | 0.20 |
|  | A5B605 | ELF5A-1 | S2 | 0.20 | 0.11 | 0.12 |
|  | D7SIZ7 | CPN20 | S124 | 0.91 | 1.46 | 1.22 |
|  |  |  | T176 | 0.72 | 0.80 | 0.48 |
|  | F6HYR3 | CDC48-related | S124 | 0.89 | 0.80 | 0.69 |
|  | A5AIE0 | not assigned | S106 | 1.21 | 1.35 | 1.29 |
| **acetylation** | A5ASW8 | LHCB6 | K219 | 1.21 | 0.45 | 0.79 |
|  | D7SNZ1 | FNR1 | K327 | 2.24 | 1.10 | 0.65 |
|  | D7TKA1 | GLO1 | K220 | 1.38 | 1.55 | 1.25 |
|  | F6H7I9 | AMT | K253 | 1.28 | 1.55 | 1.23 |
|  | P56648 | RBCL | K14 | 1.35 | 1.33 | 1.33 |
|  |  |  | K146 | 1.48 | 1.37 | 1.44 |
|  |  |  | K175 | 1.32 | 1.04 | 1.25 |
|  |  |  | K177 | 1.57 | 1.24 | 1.48 |
|  |  |  | K183 | 1.38 | 1.30 | 1.32 |
|  |  |  | K201 | 1.31 | 0.67 | 1.20 |
|  |  |  | K227 | 1.35 | 1.22 | 1.29 |
|  |  |  | K236 | 1.55 | 1.67 | 1.35 |
|  |  |  | K316 | 1.44 | 1.52 | 1.40 |
|  |  |  | K334 | 1.74 | 1.69 | 2.17 |
|  | A5C718 | RBCS1A | K70 | 1.65 | 1.42 | 1.22 |
|  |  |  | K96 | 1.26 | 1.51 | 1.37 |
|  |  |  | K105 | 1.73 | 1.86 | 1.83 |
|  |  |  | K144 | 1.35 | 1.54 | 1.42 |
|  |  |  | K178 | 1.13 | 1.40 | 1.06 |
|  | D7UDC9 | GAPB | K132 | 1.64 | 1.85 | 1.34 |
|  |  |  | K141 | 1.35 | 1.63 | 1.22 |
|  | D7SKB2 | RCA | K204 | 1.26 | 1.52 | 1.12 |
|  |  |  | K371 | 1.33 | 1.65 | 1.12 |
|  |  |  | K392 | 1.18 | 1.24 | 0.65 |
|  | F6GSG7 | GAPC2 | K261 | 0.94 | 0.84 | 0.70 |
|  | D7TWP2 | BCA1 | K86 | 1.16 | 1.38 | 1.16 |
|  |  |  | K149 | 1.34 | 1.59 | 1.25 |
|  |  |  | K188 | 1.03 | 1.32 | 1.09 |
|  |  |  | K298 | 1.25 | 1.30 | 1.22 |
|  | F6HHP1 | ADA2a | K238 | 1.04 | 0.84 | 0.55 |
|  | E0CRG0 | SAP domain- containing protein | K151 | 1.34 | 0.92 | 1.01 |
|  | A5BE97 | Probable  histone H2A.4 | K6 | 1.16 | 1.86 | 1.17 |
|  | E0CT43 | HTA1 | K6 | 1.26 | 1.50 | 1.20 |
|  |  |  | K14 | 1.47 | 1.42 | 1.07 |
|  | E0CRN7 | RPS6B | K14 | 1.60 | 2.06 | 1.42 |
|  | A5B605 | ELF5A-1 | K49 | 0.51 | 0.24 | 0.13 |
|  | D7SIZ7 | CPN20 | K159 | 1.13 | 1.33 | 1.15 |
|  | F6HYR3 | CDC48-related | K1072 | 0.60 | 0.47 | 0.44 |
|  | A5AIE0 | not assigned | K100 | 1.26 | 1.78 | 1.62 |
|  |  |  | K265 | 1.39 | 1.39 | 1.12 |

**Table S2 Phosphorylation level changes in proteins related to alternative splicing at 35^o^C, 40^o^C and 45^o^C in grape leaves**

| **Uniprot ID** | **Protein name** | **Phosphosite** | **35℃** | **40℃** | **45℃** |
| --- | --- | --- | --- | --- | --- |
| F6HKQ4 | RS2Z32 | S397 | 1.17 | 2.77 | 4.07 |
| E0CVB9 | SR34A | S191 | 0.85 | 0.96 | 1.45 |
| D7TUZ2 | SCL28 | S148 | 1.03 | 0.53 | 1.16 |
|  |  | S152 | 1.03 | 0.53 | 1.16 |
| D7TLV0 | SCL30 | S82 | 1.06 | 0.91 | 1.39 |
|  |  | S275 | 1.22 | 0.89 | 1.54 |
| D7TNS8 | SCL30A | S89 | 1.08 | 1.02 | 1.54 |
|  |  | S95 | 0.85 | 0.67 | 0.76 |
|  |  | S97 | 1.14 | 1.30 | 2.10 |
| E0CRK0 | SC35 | S187 | 0.93 | 0.66 | 0.66 |
|  |  | S230 | 1.49 | 1.09 | 2.58 |
|  |  | T232 | 1.49 | 1.09 | 2.58 |
| F6HPN0 | RSZ22A | S134 | 1.19 | 0.84 | 1.97 |
|  |  | S141 | 1.46 | 1.01 | 1.86 |
|  |  | S143 | 1.54 | 1.09 | 2.05 |
| D7T0J3 | RSZ22 | S126 | 1.25 | 0.88 | 2.08 |
|  |  | Y153 | 1.54 | 1.48 | 3.40 |
|  |  | S156 | 1.72 | 1.68 | 4.48 |
| F6HQW9 | RS2Z33 | S179 | 1.17 | 0.82 | 1.50 |
|  |  | T181 | 1.17 | 0.83 | 1.50 |
|  |  | S192 | 1.07 | 1.20 | 1.43 |
|  |  | S206 | 1.03 | 1.32 | 1.97 |
| D7UCX3 | RS31 | S167 | 1.23 | 0.96 | 1.41 |
|  |  | S172 | 1.23 | 0.96 | 1.41 |
|  |  | S174 | 1.60 | 1.43 | 2.00 |
|  |  | S183 | 0.95 | 0.71 | 0.78 |
|  |  | S207 | 0.81 | 0.68 | 0.73 |
| F6I322 | RS41 | S275 | 0.94 | 0.65 | 0.95 |
|  |  | S277 | 0.94 | 0.65 | 0.95 |
|  |  | S285 | 0.89 | 0.71 | 0.92 |
|  |  | S298 | 0.93 | 0.75 | 1.08 |
|  |  | S303 | 1.06 | 0.76 | 1.15 |
| F6HC22 | RS2Z33 | S222 | 0.82 | 0.77 | 1.08 |
|  |  | S255 | 1.11 | 0.73 | 1.59 |
|  |  | T257 | 1.11 | 0.70 | 1.53 |
|  |  | S262 | 1.13 | 0.79 | 1.74 |
|  |  | S285 | 0.77 | 0.90 | 1.03 |
|  |  | S290 | 0.91 | 0.71 | 0.89 |
|  |  | S292 | 1.05 | 1.18 | 1.77 |
|  |  | S301 | 1.10 | 1.29 | 2.04 |
| F6GUK3 | SCL30A | S5 | 1.40 | 1.39 | 2.94 |
|  |  | S7 | 1.16 | 1.07 | 2.68 |
|  |  | S9 | 1.37 | 1.33 | 2.69 |
|  |  | S164 | 1.40 | 1.09 | 1.95 |
|  |  | S166 | 1.42 | 1.08 | 1.96 |
|  |  | S188 | 1.24 | 0.89 | 1.40 |
|  |  | T190 | 1.24 | 0.89 | 1.40 |
|  |  | S225 | 0.92 | 0.67 | 0.58 |
|  |  | S227 | 1.22 | 1.55 | 2.82 |
| F6HP66 | RS41 | S188 | 0.85 | 0.67 | 1.08 |
|  |  | S197 | 1.08 | 0.74 | 0.69 |
|  |  | Y202 | 1.11 | 0.61 | 0.70 |
|  |  | S209 | 1.94 | 2.65 | 5.89 |
|  |  | S217 | 0.89 | 0.77 | 1.02 |
|  |  | S225 | 0.87 | 0.73 | 1.07 |
|  |  | S281 | 1.58 | 1.35 | 2.37 |
|  |  | S318 | 1.16 | 2.55 | 5.96 |
|  |  | S320 | 1.16 | 2.44 | 5.62 |
|  |  | S342 | 0.84 | 0.76 | 0.78 |
|  |  | S345 | 0.82 | 0.69 | 0.80 |
|  |  | T377 | 0.93 | 0.57 | 0.79 |
|  |  | S386 | 0.94 | 0.67 | 1.00 |
|  |  | S388 | 0.95 | 0.67 | 0.97 |
|  |  | S435 | 1.19 | 1.73 | 2.75 |
|  |  | S437 | 1.36 | 2.16 | 3.82 |
|  |  | S445 | 1.33 | 2.26 | 4.58 |
|  |  | S453 | 1.31 | 2.36 | 4.59 |
|  |  | S456 | 1.27 | 2.18 | 4.55 |
|  |  | S479 | 1.35 | 1.89 | 2.35 |
|  |  | S481 | 1.12 | 1.88 | 2.46 |

**Table S3 Phosphorylation and acetylation level changes in proteins related to photosynthesis at 35^o^C, 40^o^C and 45^o^C in grape leaves**

|  | **Uniprot ID** | **Protein name** | **Phosphosite /Acetylsites** | **35℃** | **40℃** | **45℃** |
| --- | --- | --- | --- | --- | --- | --- |
| **phosphorylation** | A5BAI4 | LHCB1.3 | T38 | 1.30 | 0.21 | 0.34 |
|  |  |  | S44 | 1.35 | 0.90 | 1.10 |
|  |  |  | S46 | 1.00 | 0.58 | 0.78 |
|  | F6HKS7 | LHCB4.1 | T164 | 1.04 | 0.53 | 0.61 |
|  | A5ASW8 | LHCB6 | T180 | 1.43 | 0.63 | 1.21 |
|  |  |  | T188 | 1.38 | 1.93 | 2.92 |
|  | A5AWT3 | PsbR | Y52 | 1.55 | 3.89 | 5.12 |
|  |  |  | S67 | 1.56 | 1.59 | 2.00 |
|  |  |  | Y77 | 2.80 | 5.49 | 7.95 |
|  | Q0ZIZ0 | PsbH | T3 | 0.55 | 0.18 | 0.19 |
|  |  |  | T5 | 0.56 | 0.18 | 0.19 |
|  | F6H2R3 | PsaF | S94 | 0.89 | 0.69 | 0.82 |
|  | F6I5I9 | LHCA3 | S36 | 0.65 | 0.44 | 0.64 |
|  | D7SNZ1 | FNR1 | T169 | 0.99 | 1.12 | 0.67 |
|  | A5C718 | RBCS1A | T36 | 0.55 | 0.56 | 0.58 |
|  |  |  | S117 | 0.90 | 0.88 | 1.54 |
|  |  |  | T151 | 1.06 | 1.26 | 1.49 |
|  |  |  | Y153 | 1.14 | 1.21 | 1.41 |
|  |  |  | Y177 | 0.83 | 0.75 | 0.85 |
|  |  |  | S180 | 1.31 | 1.32 | 1.48 |
|  | P56648 | RBCL | Y190 | 0.97 | 1.10 | 1.38 |
|  |  |  | S228 | 0.94 | 1.16 | 1.56 |
|  | F6HNV2 | RCA | S54 | 0.67 | 0.92 | 2.71 |
|  | D7SKB2 | RCA | S53 | 0.65 | 0.81 | 0.73 |
|  |  |  | S79 | 0.98 | 1.44 | 1.90 |
|  | D7THJ7 | RCA | S51 | 0.81 | 1.99 | 5.67 |
|  |  |  | S79 | 1.00 | 1.52 | 2.58 |
|  |  |  | S434 | 0.86 | 1.24 | 2.21 |
|  | D7UAT6 | FBA | S32 | 1.16 | 1.41 | 1.17 |
|  |  |  | T35 | 1.70 | 2.25 | 2.70 |
|  |  |  | S42 | 1.50 | 1.98 | 3.77 |
|  |  |  | S330 | 1.80 | 4.11 | 4.92 |
|  | F6GWQ0 | FBA5 | S369 | 0.94 | 1.84 | 1.81 |
|  |  |  | S382 | 1.09 | 1.32 | 1.18 |
|  | D7UDC9 | GAPB | T298 | 1.05 | 1.36 | 0.85 |
| **acetylation** | A5AE63 | LHCA1 | K183 | 0.89 | 0.46 | 0.66 |
|  | A5ASW8 | LHCB6 | K219 | 1.21 | 0.45 | 0.79 |
|  | D7ST76 | PsbD-2 | K7 | 0.91 | 0.37 | 0.58 |
|  | F6I229 | PsbO-2 | K161 | 1.34 | 1.65 | 1.31 |
|  | A5B1D3 | PsbP1 | K107 | 1.30 | 1.45 | 1.18 |
|  | F6H8B4 | PsbQ2 | K117 | 1.12 | 1.31 | 1.24 |
|  |  |  | K145 | 1.25 | 1.85 | 1.43 |
|  |  |  | K205 | 1.24 | 1.54 | 1.11 |
|  | A5BHE6 | PsaH2 | K98 | 1.23 | 1.41 | 1.23 |
|  | Q0ZJ35 | ATPA | K114 | 1.11 | 0.73 | 0.90 |
|  | Q0ZJ13 | ATPB | K225 | 0.74 | 0.70 | 0.79 |
|  |  |  | K495 | 1.37 | 1.59 | 1.05 |
|  | D7SNZ1 | FNR1 | K327 | 2.24 | 1.10 | 0.65 |
|  | A5C718 | RBCS1A | K70 | 1.65 | 1.42 | 1.22 |
|  |  |  | K96 | 1.26 | 1.51 | 1.37 |
|  |  |  | K105 | 1.73 | 1.86 | 1.83 |
|  |  |  | K144 | 1.35 | 1.54 | 1.42 |
|  |  |  | K178 | 1.13 | 1.40 | 1.06 |
|  | P56648 | RBCL | K14 | 1.35 | 1.33 | 1.33 |
|  |  |  | K146 | 1.48 | 1.37 | 1.44 |
|  |  |  | K175 | 1.32 | 1.04 | 1.25 |
|  |  |  | K177 | 1.57 | 1.24 | 1.48 |
|  |  |  | K183 | 1.38 | 1.30 | 1.32 |
|  |  |  | K201 | 1.31 | 0.67 | 1.20 |
|  |  |  | K227 | 1.35 | 1.22 | 1.29 |
|  |  |  | K236 | 1.55 | 1.67 | 1.35 |
|  |  |  | K316 | 1.44 | 1.52 | 1.40 |
|  |  |  | K334 | 1.74 | 1.69 | 2.17 |
|  | D7SKB2 | RCA | K204 | 1.26 | 1.52 | 1.12 |
|  |  |  | K371 | 1.33 | 1.65 | 1.12 |
|  |  |  | K392 | 1.18 | 1.24 | 0.65 |
|  | A5BDH7 | FBA | K60 | 1.05 | 1.32 | 1.10 |
|  |  |  | K292 | 1.09 | 1.31 | 1.13 |
|  | A5CAF8 | PGK | K88 | 1.15 | 1.32 | 1.07 |
|  |  |  | K146 | 0.79 | 0.49 | 0.26 |
|  |  |  | K197 | 0.85 | 0.87 | 0.76 |
|  |  |  | K208 | 0.90 | 0.87 | 0.75 |
|  |  |  | K278 | 1.57 | 1.47 | 1.35 |
|  |  |  | K414 | 1.03 | 0.94 | 0.76 |
|  | D7UDC9 | GAPB | K132 | 1.64 | 1.85 | 1.34 |
|  |  |  | K141 | 1.35 | 1.63 | 1.22 |
|  | F6I134 | TIM | K180 | 1.14 | 1.39 | 1.18 |
|  | F6HAM6 | TKT | K71 | 0.89 | 0.88 | 0.74 |
|  |  |  | K304 | 0.77 | 0.62 | 0.70 |
|  |  |  | K387 | 0.83 | 0.75 | 0.74 |
|  | D7TMH3 | SBPASE | K304 | 1.71 | 2.27 | 0.90 |
|  | F6HTS6 | Rib5P | K62 | 1.10 | 1.57 | 1.35 |
|  | F6H7Q2 | PRK | K407 | 0.79 | 0.70 | 0.68 |
|  | F6GT65 | RPE | K163 | 1.68 | 1.74 | 1.10 |
|  | F6GWA8 | CPN60A | K270 | 1.51 | 1.85 | 1.61 |

**Table S4 Phosphorylation and acetylation level changes in heat shock proteins at 35^o^C, 40^o^C and 45^o^C in grape leaves**

|  | **Uniprot ID** | **Protein name** | **Phosphosite /Acetylsites** | **35℃** | **40℃** | **45℃** |
| --- | --- | --- | --- | --- | --- | --- |
| **phosphorylation** | F6HNM6 | HSP17.4A | S113 | 1.25 | 1.28 | 1.98 |
|  | F6H3Q4 | HSP17.6 | S29 | 0.91 | 2.51 | 2.59 |
|  | A5AQ47 | HSP17.6 | S29 | 0.91 | 2.75 | 3.19 |
|  | F6HNM7 | HSP17.6C | S10 | 1.87 | 1.60 | 1.38 |
|  |  |  | S154 | 1.54 | 7.26 | 9.88 |
|  | D7T1L1 | HSP20 | S241 | 0.63 | 0.63 | 0.54 |
|  | D7UDS4 | HSP20 | S140 | 0.69 | 0.65 | 0.81 |
|  | D7TN47 | HSP23.6 | S12 | 1.38 | 9.22 | 7.62 |
|  | A5AFX4 | HSP23.6 | S27 | 1.03 | 5.84 | 8.13 |
|  |  |  | S56 | 1.17 | 4.76 | 9.17 |
|  | D7TAL0 | HSP25.3 | T3 | 0.80 | 0.74 | 0.66 |
|  | D7U4L8 | HSP40 | S321 | 1.14 | 0.85 | 2.86 |
|  | A5ALT5 | HSP40.3 | S25 | 1.72 | 1.94 | 2.55 |
|  | D7T3A9 | HSP40.3 | S25 | 1.57 | 1.67 | 2.17 |
|  | F6I6Y7 | HSP40.10 | S345 | 0.72 | 0.61 | 0.54 |
|  |  |  | S349 | 0.74 | 0.61 | 0.56 |
|  | F6HNX5 | HSP70.1 | S82 | 1.13 | 0.96 | 0.74 |
|  |  |  | S195 | 0.89 | 1.25 | 1.42 |
|  |  |  | S533 | 1.24 | 1.57 | 1.64 |
|  | F6H6C3 | HSP81.1 | S224 | 1.17 | 2.38 | 1.79 |
|  | F6HCU9 | HSP90.2 | S525 | 1.10 | 1.76 | 2.44 |
| **acetylation** | F6GTP0 | HSP70.1 | K613 | 0.88 | 0.99 | 0.70 |
|  | A5C0Z3 | HSP70.6 | K191 | 1.31 | 1.49 | 1.19 |
